# Parkinson’s Disease: From Gene–Environment Risk to Precision Therapy

**DOI:** 10.3390/medsci14010072

**Published:** 2026-02-05

**Authors:** Oscar Arias-Carrión

**Affiliations:** 1 División de Neurociencias Clínica, Instituto Nacional de Rehabilitación Luis Guillermo Ibarra Ibarra, Mexico City 14389, Mexico; ariasemc2@gmail.com; 2 Tecnologico de Monterrey, Escuela de Medicina y Ciencias de la Salud, Mexico City 14380, Mexico

**Keywords:** Parkinson’s disease, α-synucleinopathy, gene–environment interaction, biomarkers, disease modification, precision medicine, health equity

## Abstract

Parkinson’s disease (PD) is a progressive and heterogeneous neurodegenerative disorder and one of the fastest-growing causes of neurological disability worldwide. Although historically defined by motor manifestations resulting from nigrostriatal dopaminergic degeneration, PD is now recognized as a multisystem disorder. Non-motor features—including autonomic dysfunction, neuropsychiatric symptoms, cognitive impairment, and sleep-related disorders—frequently precede motor onset by years or even decades, delineating a clinically meaningful prodromal phase. The aetiology of PD reflects a complex interplay between genetic susceptibility and environmental exposures. Approximately 20% of cases are linked to identifiable pathogenic variants, most commonly in *LRRK2*, *GBA1*, and *SNCA*, whereas the majority arise from cumulative interactions among environmental factors, lifestyle determinants, and common genetic risk variants rather than from single causal mechanisms. Despite substantial advances in understanding disease biology, current therapies remain fundamentally symptomatic. Dopaminergic pharmacotherapy and device-aided interventions improve motor function and, in selected contexts, functional outcomes, but they do not modify disease progression. Non-motor symptoms remain a dominant driver of disability and reduced quality of life. Recent conceptual frameworks propose redefining PD as a biologically defined α-synucleinopathy. Emerging biomarkers, including α-synuclein seed amplification assays in cerebrospinal fluid and peripheral tissues, offer unprecedented opportunities to define biological disease, enable early detection, and stratify patients. However, biomarker positivity currently informs diagnosis and classification rather than prognostication or therapeutic selection, and validated intermediate endpoints linking biomarker change to sustained functional benefit remain lacking. Consequently, translation into disease-modifying therapies has been constrained by late-stage intervention, reliance on clinically defined populations, limited trial generalizability, and marked global inequities in access to advanced diagnostics and treatments. This narrative review synthesizes current evidence on PD epidemiology, diagnosis, aetiology, progression, and treatment, emphasizing gene–environment interactions, convergence on shared pathogenic pathways, limitations of existing therapeutic paradigms, and the as-yet unrealized potential of biologically informed precision care.

## 1. Introduction

Parkinson’s disease (PD) is a common, chronic, and progressive neurodegenerative disorder that predominantly affects individuals in later life and represents one of the fastest-growing causes of neurological disability worldwide [1,2]. Clinically, PD is defined by cardinal motor symptoms—bradykinesia, rigidity, resting tremor, and postural instability—that typically emerge asymmetrically and reflect dysfunction of the nigrostriatal dopaminergic system [3]. Pathologically, the disease is characterized by selective neuronal loss and the intraneuronal accumulation of misfolded α-synuclein aggregates, forming Lewy bodies and Lewy neurites, which involve both the central and peripheral nervous systems, including the brainstem, limbic regions, neocortex, and the autonomic nervous system [4,5].

**Figure 1:**
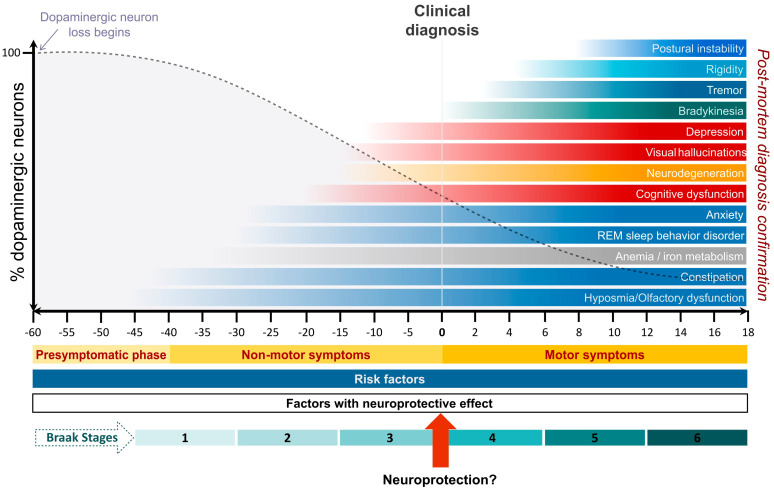
Temporal dissociation between neurodegeneration, clinical phenotypes, and diagnostic thresholds in Parkinson’s disease. This conceptual framework depicts PD as a prolonged, biologically progressive disorder in which neurodegeneration, symptom expression, and clinical diagnosis are temporally uncoupled. Time is shown relative to clinical diagnosis (year 0), spanning presymptomatic, non-motor–predominant, and motor-dominant phases. The vertical axis represents relative integrity of the nigrostriatal dopaminergic system rather than absolute neuronal counts. The dashed trajectory illustrates a schematic pattern of dopaminergic decline and does not imply fixed quantitative thresholds or uniform rates of progression. Non-motor features—including hyposmia, constipation, rapid eye movement (REM) sleep behaviour disorder, anxiety, depression, cognitive dysfunction, hallucinations, and disturbances of iron metabolism—emerge early, often decades before diagnosis, reflecting early involvement of extranigral, autonomic, limbic, and brainstem networks. These manifestations frequently contribute substantially to functional impairment and reduced quality of life but remain under-represented in motor-centred diagnostic frameworks. In contrast, cardinal motor features—bradykinesia, tremor, rigidity, and postural instability—typically arise later, coinciding with advanced nigrostriatal dysfunction and the point at which clinical diagnosis is usually established. By this stage, a large proportion of dopaminergic neurodegeneration has already occurred, constraining the potential impact of disease-modifying or neuroprotective interventions. Braak neuropathological stages are schematically shown to contextualize canonical patterns of α-synuclein propagation, while acknowledging substantial biological heterogeneity, genetic divergence, and incomplete clinicopathological concordance. Risk factors and factors associated with relative resilience are depicted as acting across the disease continuum, highlighting that vulnerability and resilience are shaped long before clinical recognition. The indicated window emphasizes the theoretical opportunity for neuroprotective intervention, underscoring a central challenge in PD: the persistent misalignment between the timing of neurodegeneration, the emergence of clinically actionable phenotypes, and the thresholds at which intervention is currently initiated.

Over the past two decades, PD has been reconceptualized as a multisystem disorder extending far beyond its classic motor phenotype (Figure 1). Non-motor symptoms—including hyposmia, rapid eye movement sleep behaviour disorder, autonomic dysfunction, mood disturbances, and cognitive impairment—are now recognized as integral components of the disease and often precede motor symptom onset by years or even decades [6,7,8]. This prodromal phase challenges traditional clinicopathological paradigms and underscores the limitations of diagnosing PD solely on motor criteria [3,5,7]. Importantly, prodromal features are individually nonspecific and acquire clinical relevance only when interpreted within probabilistic frameworks or integrated with biological markers, with implications for early detection, staging, and trial design rather than routine diagnosis [9].

The aetiology of PD is complex and heterogeneous, arising from the interplay between genetic susceptibility and environmental exposures. Approximately 20% of cases are associated with identifiable pathogenic variants, most commonly involving *LRRK2*, *GBA1*, and *SNCA*, while the majority of PD cases are sporadic [10,11]. These genetic forms have provided critical insights into disease-relevant biological pathways, including lysosomal dysfunction, impaired protein degradation, mitochondrial failure, and neuroinflammation [12,13]. However, heritability estimates of approximately 20% to 30% indicate that genetic susceptibility alone is insufficient to explain disease emergence, highlighting the contribution of environmental factors acting on a background of common genetic risk [14,15].

Among environmental exposures, pesticides and chlorinated solvents have been most consistently linked to increased PD risk, often in a dose-dependent manner [16,17,18]. Additional risk factors include traumatic brain injury and certain metabolic and inflammatory conditions, whereas lifestyle factors such as cigarette smoking, caffeine consumption, and regular physical activity appear inversely associated with PD risk [19]. Although these associations are robust at the epidemiological level, their biological effects converge on shared pathogenic pathways—mitochondrial dysfunction, oxidative stress, impaired autophagy, immune dysregulation, and α-synuclein aggregation—rather than representing isolated causal mechanisms, supporting the view of PD as a disorder of gene–environment interaction rather than a single-pathway disease entity [15].

Despite major advances in understanding PD pathophysiology, no therapy has yet been shown to alter disease progression. Current pharmacological treatments—principally dopaminergic therapies—remain focused on symptomatic relief and are limited by diminishing efficacy, motor complications, and neuropsychiatric adverse effects over time [20,21]. Deep brain stimulation offers substantial benefit for motor fluctuations in selected patients but does not meaningfully address non-motor symptoms or halt neurodegeneration [22,23]. The repeated failure of neuroprotective trials underscores a structural limitation of current paradigms: interventions are typically initiated after substantial neuronal loss and within clinically defined populations that inadequately capture underlying biological heterogeneity, highlighting the urgency of identifying biologically defined disease states earlier in the disease course [9,24].

Recent conceptual advances, therefore, propose redefining PD as a biologically defined α-synucleinopathy rather than a purely clinical syndrome [4,9]. Emerging biomarkers—including α-synuclein seed amplification assays in cerebrospinal fluid, skin, and peripheral tissues—demonstrate high sensitivity and specificity for synucleinopathies and may enable the identification of PD at prodromal or preclinical stages [25,26,27]. However, biomarker positivity currently supports biological classification and trial stratification rather than reliable prognostication or treatment selection, and validated intermediate endpoints linking biomarker change to sustained functional benefit remain unavailable [9,28].

Against this backdrop, the central aim of this review is to examine why expanding knowledge of gene–environment interactions, biological heterogeneity, and biomarker development has not yet translated into effective, equitable precision therapy for PD. This narrative review synthesizes current evidence on PD epidemiology, diagnosis, aetiology, progression, and treatment, with particular emphasis on the interaction between genetic architecture and environmental exposure, the functional limitations of existing therapeutic paradigms, and the emerging—but still aspirational—role of biological classification. By identifying critical mechanistic, clinical, and implementation gaps, we aim to reframe future directions toward prevention, early biological definition, patient-centred functional outcomes, and precision care that is both evidence-based and globally scalable, ultimately reshaping the trajectory of PD care.

## 2. Epidemiology

PD demonstrates a pronounced age-related increase in both incidence and prevalence, with men affected approximately twice as often as women [1,29]. Population-based studies from North America report incidence rates ranging from 47 to 77 cases per 100,000 individuals aged 45 years and older, and from 108 to 212 cases per 100,000 individuals aged 65 years and older, underscoring the dominant influence of ageing on disease risk [2,29]. These demographic patterns are consistent across multiple regions and reinforce ageing as the strongest non-modifiable risk factor for PD.

Epidemiological data indicate variability in reported PD incidence across racial and ethnic groups, with higher diagnosed incidence among White populations compared with Black or Asian populations [2,29]. However, such differences must be interpreted with caution. Neuropathological studies demonstrate that the prevalence of Lewy body pathology—a defining feature of PD—is comparable between Black and White individuals at autopsy, suggesting that disparities in diagnosed incidence are more likely to reflect differences in access to specialist care, diagnostic practices, health-system engagement, and survival, rather than true biological differences in disease susceptibility [30]. This discrepancy highlights the extent to which structural and healthcare-related factors shape epidemiological estimates of PD.

The prevalence of PD in the United States is estimated at approximately 572 cases per 100,000 individuals aged 45 years and older, with prevalence increasing sharply with advancing age [1]. Mortality among individuals with PD remains substantially elevated, with age- and sex-adjusted mortality rates approximately 60% higher than those observed in the general population, reflecting both disease-related complications and comorbid conditions [1]. These excess mortality rates underscore the cumulative impact of motor disability, non-motor symptoms, and systemic complications over the disease course.

Beyond its clinical impact, PD imposes a substantial and rapidly growing economic burden. In the United States, the total annual cost of PD—including direct medical expenditures and indirect costs related to lost productivity and caregiving—was estimated at $52 billion in 2017 and is projected to rise to $79 billion by 2037, driven largely by population ageing and increasing disease prevalence [31]. Importantly, these estimates do not fully capture the disproportionate burden borne by caregivers or the downstream costs associated with delayed diagnosis and fragmented care. Collectively, these trends underscore the urgent need for strategies focused on earlier disease identification, biologically informed risk stratification, prevention where feasible, and health-system approaches to mitigate long-term disability and inequity (Figure 1).

## 3. Diagnosing Parkinson’s Disease

PD, historically defined by its motor manifestations resulting from nigrostriatal dopaminergic neuron loss, is now recognized as a complex multisystem neurological disorder (Figure 1). Non-motor symptoms constitute a core component of the disease and include sleep-related disorders, cognitive impairment, neuropsychiatric symptoms, autonomic dysfunction—such as constipation, urogenital dysfunction, and orthostatic hypotension—and sensory abnormalities, including hyposmia and pain [6,7,8]. Significantly, several non-motor features, particularly hyposmia and rapid eye movement (REM) sleep behaviour disorder—characterized by loss of normal muscle atonia during REM sleep with dream-enactment behaviours—may precede the onset of motor symptoms by many years, defining a prodromal phase of PD [6,7,8]. Although individually nonspecific, the accumulation and temporal patterning of these features provide probabilistic information regarding disease risk and progression, and their gradual progression contributes substantially to disability and functional decline over the disease course.

To standardize diagnosis, the International Parkinson and Movement Disorder Society (MDS) has established clinical diagnostic criteria for PD and research criteria for prodromal PD [6,8]. These frameworks rely primarily on clinical features, with ancillary investigations used when diagnostic uncertainty exists. Importantly, the MDS prodromal criteria are not intended for routine clinical diagnosis but for risk stratification in research settings, integrating multiple nonspecific features into a probabilistic model. Although no imaging modality can definitively confirm PD, functional imaging of the presynaptic dopaminergic system—using ^123I-ioflupane single-photon emission computed tomography (SPECT) or ^18F-fluorodopa positron emission tomography (PET)—is valuable for differentiating PD from conditions such as essential tremor or drug-induced parkinsonism [32]. Meta-analytic data indicate that ^123I-ioflupane SPECT imaging demonstrates sensitivity and specificity exceeding 90% and can lead to changes in diagnosis in approximately one-third of cases and alterations in clinical management in more than half of patients evaluated [32]. However, these modalities assess dopaminergic dysfunction rather than disease aetiology and do not distinguish PD from other degenerative parkinsonian syndromes [33].

Structural magnetic resonance imaging (MRI) plays an important complementary role by identifying features suggestive of alternative neurodegenerative parkinsonian disorders [33,34]. Characteristic changes involving the basal ganglia or infratentorial structures may point toward diagnoses such as progressive supranuclear palsy or multiple system atrophy, for which specific MDS diagnostic criteria exist [33,35,36]. Ongoing advances in MRI techniques, including quantitative and high-field imaging, may further enhance diagnostic accuracy and facilitate earlier differentiation among parkinsonian syndromes [34,37]. Nevertheless, MRI remains primarily an exclusionary and pattern-recognition tool rather than a definitive diagnostic test for PD.

Neuropathologically, PD is defined by the intraneuronal accumulation of misfolded α-synuclein, forming Lewy bodies and Lewy neurites, which are detected in up to 90% of clinically diagnosed cases at autopsy [38]. This pathology affects a characteristic network of regions, including brainstem nuclei—such as the dorsal motor nucleus of the vagus, locus coeruleus, and substantia nigra—the peripheral autonomic nervous system, and limbic and neocortical areas [38]. A defining feature is the marked loss of pigmented, dopamine-producing neurons in the substantia nigra. However, α-synuclein pathology is neither uniformly distributed nor universally present across all genetic and clinical subtypes, underscoring biological heterogeneity within the PD spectrum.

Despite established clinical criteria, diagnostic accuracy remains imperfect, particularly in early disease stages. Clinicopathological studies have demonstrated concordance rates as low as 28% between initial clinical diagnosis and autopsy findings, although accuracy improves substantially with longer disease duration, reaching approximately 89% in advanced stages [39]. Diagnostic precision is highest when evaluations are conducted by movement disorder specialists, highlighting the importance of expert clinical assessment in PD diagnosis [39]. Collectively, these limitations highlight the need for biologically informed diagnostic frameworks that complement, rather than replace, expert clinical judgment.

## 4. Etiology of Parkinson’s Disease

PD is a complex and heterogeneous disorder arising from the interplay between genetic susceptibility and environmental exposures. Approximately 20% of PD cases are attributable to identifiable pathogenic genetic variants, collectively referred to as monogenic PD [10,11]. Among autosomal dominant forms with incomplete penetrance, mutations in *LRRK2* are the most prevalent, accounting for approximately 1% to 2% of all PD cases and up to 40% of familial cases in specific populations [11]. Variants in *GBA1*, which encodes the lysosomal enzyme glucocerebrosidase, are present in approximately 5% to 15% of PD cases, with particularly high prevalence among individuals of Ashkenazi Jewish and North African ancestry [10]. Less common dominant mutations, including those in *SNCA* and *VPS35*, account for fewer than 1% of cases [11].

Autosomal recessive forms of PD, most commonly caused by mutations in *PRKN*, *PINK1*, and *DJ1*, are typically associated with early-onset disease and distinct clinical phenotypes [11]. Although rare, these variants represent the most common genetic causes of PD in younger patients. Pathologically, abnormal α-synuclein accumulation is a defining feature of PD and is commonly observed in cases associated with *SNCA* and *GBA1* mutations, as well as in approximately half of *LRRK2*-associated cases [10]. In contrast, recessive forms of PD often exhibit minimal or absent α-synuclein pathology, fewer non-motor symptoms, and more prominent dystonia, illustrating that neurodegeneration in PD can arise through partially overlapping but biologically distinct mechanisms [11]. These differences underscore that α-synucleinopathy, while central, is not uniformly expressed across all genetic subtypes.

Beyond monogenic forms, genome-wide association studies have substantially expanded the genetic architecture of PD by identifying more than 90 risk loci, each conferring modest individual effects [10]. Many of these loci cluster near known causative genes and implicate convergent biological pathways, including lysosomal function, mitochondrial homeostasis, immune regulation, and synaptic biology. However, most genetic studies have disproportionately focused on populations of European ancestry, limiting generalisability. Recent studies in underrepresented populations have identified population-specific risk variants, including a *GBA1* variant accounting for approximately 39% of PD cases among individuals of African ancestry, highlighting the importance of inclusive global genetic research for accurate risk estimation and biological inference [40].

In individuals without high-penetrance mutations, heritability estimates for PD range from 20% to 30%, indicating a substantial contribution from environmental factors [16]. Identifying specific environmental risks has proven challenging due to methodological limitations, exposure misclassification, and the multifactorial nature of disease causation. Epidemiological studies have often examined individual exposures in isolation, despite growing evidence that PD reflects the cumulative burden of multiple environmental insults acting on a genetically susceptible background over time [15].

Among environmental factors, exposure to pesticides and industrial solvents has been most consistently associated with increased PD risk. Residential or occupational exposure to agents such as paraquat, rotenone, 2,4-dichlorophenoxyacetic acid, and chlorinated solvents—including trichloroethylene and perchloroethylene—has been linked to dose-dependent increases in PD risk, frequently exceeding 40% [16,17,18]. Experimental models corroborate these epidemiological findings, demonstrating that these toxicants induce mitochondrial dysfunction, oxidative stress, impaired proteostasis, neuroinflammation, and selective dopaminergic vulnerability, thereby providing mechanistic plausibility for observed population-level associations [12].

Additional environmental and lifestyle factors may further modify PD risk (Figure 1). High consumption of dairy products has been associated with increased PD risk, potentially mediated by higher brain concentrations of organochlorine compounds such as heptachlor epoxide [41]. Traumatic brain injury has also been linked to elevated risk of PD and related synucleinopathies, with reported risk increases ranging from modest to several-fold depending on injury severity and timing [19]. Other proposed risk factors—including exposure to metals, type 2 diabetes mellitus, inflammatory conditions, and infections—have shown less consistent associations across studies [15]. Importantly, many of these exposures are thought to influence disease risk through long-lasting effects on immune regulation, metabolic stress responses, and neuronal resilience rather than solely through direct neurotoxicity.

Conversely, several lifestyle factors appear to confer protection against PD. Cigarette smoking, caffeine consumption, and regular physical activity have each been associated with reduced disease risk and improved outcomes [42]. Mechanistic studies suggest that these associations may be mediated through modulation of nicotinic acetylcholine and adenosine A2A receptors, attenuation of neuroinflammatory responses, enhancement of mitochondrial efficiency, promotion of autophagy, and increased neurotrophic signalling. Converging evidence from genetic, environmental, and sporadic PD indicates that diverse risk and protective factors ultimately converge on shared biological pathways, including neuroinflammation, immune dysregulation, oxidative stress, mitochondrial dysfunction, impaired autophagy, protein aggregation, and endolysosomal system failure [12,13].

Emerging evidence further suggests that epigenetic mechanisms—including DNA methylation changes, histone modifications, chromatin remodelling, and non-coding RNAs—may mediate the interface between genetic susceptibility and environmental exposure [43], providing a biologically plausible explanation for how transient or cumulative environmental insults exert long-term effects on disease risk and progression. Together, these findings support a model of PD aetiology in which genetic architecture, environmental exposures, and epigenetic regulation interact across the lifespan to shape vulnerability, resilience, and clinical heterogeneity (Figure 1).

## 5. Parkinson’s Disease Progression

The clinical progression of PD is characterized by a dynamic, heterogeneous combination of motor and non-motor manifestations, with substantial interindividual variability in age at onset, symptom severity, and progression rate. Motor symptoms—including bradykinesia, rigidity, tremor, and postural instability—typically begin asymmetrically and gradually evolve to bilateral involvement as neurodegeneration advances [3,5]. Over time, many patients develop significant functional impairment driven by worsening motor disability, gait and balance disturbances, cognitive decline, and an increased risk of falls and fractures, although the tempo and dominant contributors to disability vary widely across individuals [1,44]. This heterogeneity limits the prognostic value of purely clinical staging systems.

Non-motor symptoms frequently precede the onset of motor dysfunction by years or even decades, reflecting early involvement of non-dopaminergic and extranigral systems. Common prodromal features include hyposmia, autonomic dysfunction, and rapid eye movement (REM) sleep behaviour disorder, which are now recognized as strong clinical markers of increased PD risk [6,7,8]. However, these features remain individually nonspecific and acquire prognostic relevance only when aggregated or integrated with biological markers. As the disease progresses, additional autonomic disturbances—such as orthostatic hypotension, impaired gastrointestinal motility, urinary dysfunction, erectile dysfunction, and altered thermoregulation—often emerge and tend to worsen over time, contributing substantially to morbidity, caregiver burden, and reduced quality of life [8].

Cognitive dysfunction represents a major determinant of long-term disability in PD. Subtle deficits in executive function, attention, and visuospatial processing may be detectable early and can precede motor symptoms in some individuals [45]. Longitudinal studies suggest that approximately 10% of patients with PD develop mild cognitive impairment or PD dementia annually, with cumulative risk increasing with disease duration [44,46]. Dementia with Lewy bodies, a closely related synucleinopathy, is characterized by early and prominent cognitive and neuropsychiatric symptoms—including visual hallucinations—in conjunction with parkinsonism and may represent either a clinical variant within the PD spectrum or a partially distinct entity with overlapping pathology [47]. These overlapping phenotypes illustrate the limitations of purely clinical boundaries within synucleinopathies.

Neuropathological studies reveal substantial overlap between PD and Alzheimer’s disease–related pathology. Alzheimer-type changes are present in approximately 38% of clinically diagnosed PD cases and in up to 89% of dementia with Lewy bodies cases, highlighting the contribution of mixed proteinopathies to cognitive decline and disease progression [48]. Such pathological convergence provides a biological explanation for divergent clinical trajectories among patients with similar motor phenotypes and underscores the need for biologically informed classification systems.

Efforts to define clinical subtypes of PD with distinct trajectories of progression have yielded inconsistent and poorly reproducible results across cohorts [49]. This lack of reproducibility likely reflects the dominance of late-stage, symptom-based phenotyping rather than accurate biological stratification. In contrast, emerging evidence suggests that genetically and biologically defined subgroups may offer greater prognostic value. For example, patients carrying *GBA1* variants exhibit a higher risk of early cognitive decline and more rapid disease progression, whereas individuals with *PRKN* mutations often demonstrate slower progression and relative sparing of cognitive function [10,11]. These observations support the concept that progression in PD is shaped by underlying biological heterogeneity rather than by a single, uniform disease course. Continued refinement of biologically informed classification systems is therefore expected to improve prognostication, patient counselling, and stratification in clinical trials.

## 6. Non-Motor Symptoms of Parkinson’s Disease

PD is increasingly recognized as a multisystem neurodegenerative disorder in which non-motor symptoms constitute core manifestations rather than secondary consequences of motor dysfunction (Table 1). These symptoms often precede the onset of classical motor features by years and progressively dominate the clinical course, shaping disability, quality of life, and prognosis (Figure 1). Although individually heterogeneous and often nonspecific, their cumulative burden and temporal evolution provide critical insight into disease biology and progression. The diversity of non-motor symptoms reflects widespread degeneration across central and peripheral nervous system networks, involving autonomic, sensory, sleep–wake, neuropsychiatric, and cognitive systems, and implicating neurotransmitter systems extending well beyond dopamine, including serotonergic, noradrenergic, and cholinergic pathways [7,50].

Autonomic dysfunction represents one of the earliest and most pervasive non-motor domains. Gastrointestinal symptoms—particularly constipation—often emerge decades before diagnosis and are linked to α-synuclein pathology in the enteric nervous system and the dorsal motor nucleus of the vagus, supporting gut–brain axis models of disease propagation [51,52]. Cardiovascular autonomic failure, including orthostatic hypotension and impaired heart rate variability, reflects sympathetic denervation and baroreflex dysfunction and contributes substantially to falls, syncope, and mortality [7,53]. Genitourinary, sexual, thermoregulatory, and cutaneous manifestations further underscore that PD involves peripheral autonomic structures early, systemically, and often independently of motor severity.

Sensory alterations form another prominent component of the non-motor phenotype. Olfactory dysfunction is among the most robust prodromal features of PD, reflecting early Lewy pathology in the olfactory bulb and limbic regions and correlating with subsequent cognitive decline, REM sleep behaviour disorder, and neuropsychiatric symptoms [7,54]. While hyposmia lacks diagnostic specificity in isolation, its presence within defined clinical or biological contexts enriches risk stratification. Visual disturbances arise from combined retinal dopaminergic loss and cortical dysfunction and contribute to falls, hallucinations, and impaired visuospatial processing. Pain and paresthesias, often preceding motor onset, reflect degeneration across nociceptive, limbic, spinal, and peripheral nerve pathways and reinforce the systemic nature of PD [55].

Sleep-related disorders provide a unique window into early neurodegeneration. REM sleep behaviour disorder (RBD) is among the most specific prodromal markers of synucleinopathies, with longitudinal studies demonstrating high conversion rates to PD and related disorders [7,8,56]. Nevertheless, RBD represents a probabilistic risk marker rather than a deterministic precursor, and its predictive value is maximized when combined with biological biomarkers [56]. Insomnia, excessive daytime sleepiness, restless legs syndrome, and obstructive sleep apnoea reflect degeneration of brainstem monoaminergic nuclei, hypothalamic arousal systems, and circadian regulatory circuits [56]. Beyond their symptomatic burden, sleep disturbances may impair glymphatic clearance of misfolded proteins, potentially accelerating α-synuclein accumulation and disease progression [4].

Within this multisystem landscape, neuropsychiatric symptoms emerge as a central and conceptually revealing domain. Depression, anxiety, apathy, hallucinations, and related affective and perceptual disturbances frequently arise in the prodromal or early stages and exert a disproportionate impact on quality of life, caregiver burden, healthcare utilization, and survival. Crucially, these symptoms are not merely psychological reactions to chronic disability but reflect intrinsic degeneration of limbic, paralimbic, and associative neural networks [44].

Depression affects approximately one-third to nearly half of individuals with PD and often precedes motor onset [57]. Parkinson’s-related depression is clinically distinct, characterized by anhedonia, irritability, psychomotor slowing, and apathy rather than pervasive sadness. Neurobiologically, it reflects degeneration of mesolimbic dopaminergic projections, serotonergic raphe nuclei, noradrenergic locus coeruleus neurons, and limbic structures such as the amygdala and thalamus [51,58]. Structural and functional imaging studies demonstrate disrupted frontolimbic connectivity, underscoring that mood disturbances arise from distributed network dysfunction rather than isolated neurotransmitter deficits.

Anxiety is similarly prevalent and clinically consequential, often coexisting with depression but representing a partially independent phenotype. Anxiety disorders in PD include generalized anxiety, panic disorder, and social anxiety, frequently fluctuating with motor states and autonomic instability [59]. Neuroimaging studies implicate altered amygdala reactivity, impaired prefrontal regulation, and salience network dysfunction, while emerging evidence links anxiety to α-synuclein pathology, oxidative stress, and inflammatory mechanisms [60]. These associations further support a shared biological substrate underlying motor, autonomic, and affective symptoms.

Apathy is one of the most disabling neuropsychiatric syndromes in PD and is distinct from both depression and cognitive impairment. Characterized by diminished motivation, emotional blunting, and reduced goal-directed behaviour, apathy reflects disruption of mesocorticolimbic circuits involving the ventral tegmental area, the nucleus accumbens, the orbitofrontal cortex, and the anterior cingulate cortex [61]. Its presence is strongly associated with cognitive decline and progression to dementia, positioning apathy as both a major determinant of disability and a marker of adverse disease trajectory rather than a secondary behavioural complication.

Psychotic symptoms, particularly visual hallucinations, mark a critical inflection point in the neuropsychiatric trajectory of PD. These symptoms arise from convergent cholinergic degeneration, dysfunction of the visual association cortex, and impaired thalamocortical filtering and are strongly associated with cognitive impairment and institutionalization [44]. Less common phenomena such as phantosmia further illustrate disruption of predictive coding within sensory–limbic networks and reinforce the breadth of perceptual dysfunction in PD.

In summary, non-motor symptoms reveal PD as a distributed, network-level disorder in which neuropsychiatric and autonomic manifestations occupy a central position. Incorporating non-motor symptom trajectories into diagnostic frameworks, biomarker research, and therapeutic trial design is essential for advancing biologically informed and patient-centred precision medicine approaches, and for aligning clinical care with both the biological reality and lived experience of PD [7].

## 7. Treatment of Parkinson’s Disease

### 7.1. Rethinking Treatment Effectiveness in Parkinson’s Disease

PD is a progressive neurodegenerative disorder in which disability emerges from the dynamic interaction between motor impairment, non-motor symptoms, cognitive decline, and environmental context (Figure 1). Traditionally, treatment success has been defined by improvement in motor signs, particularly bradykinesia and rigidity. However, large longitudinal studies and randomized trials demonstrate that motor improvement alone poorly predicts long-term functional independence, participation, or quality of life [7,20,50].

In this review, treatment effectiveness is defined primarily by the ability to stabilize daily functioning, attenuate disability progression, and preserve quality of life, rather than by isolated reductions in motor scores, OFF time, or dopaminergic responsiveness. This framework explicitly recognizes PD as a multisystem disorder and aligns therapeutic goals with patient-centred outcomes, an approach increasingly advocated in contemporary clinical research and care models [62]. Importantly, this definition does not imply neurobiological disease modification, but rather clinically meaningful functional stabilization.

### 7.2. Disease Progression and the Elusive Goal of Neuroprotection

A disease-modifying therapy is one that alters the underlying neurodegenerative process (Figure 1). Despite extensive investigation, no pharmacological treatment has convincingly demonstrated disease-modifying effects in PD. Early enthusiasm for monoamine oxidase–B inhibitors was tempered by subsequent trials demonstrating that apparent benefits reflected symptomatic dopaminergic effects rather than slowed neurodegeneration [24].

A major biological limitation is the advanced neuronal loss present at the time of clinical diagnosis. Neuropathological and imaging studies indicate that by the onset of cardinal motor symptoms, approximately two-thirds of nigrostriatal dopaminergic neurons are already dysfunctional or lost [63]. This recognition has driven interest in intervention during prodromal or biologically defined stages, supported by biomarkers of α-synuclein pathology. However, clinical trials targeting α-synuclein aggregation, propagation, or clearance—including studies in genetically enriched populations such as *GBA1* and *LRRK2* mutation carriers—have thus far yielded mixed or negative results [28,64].

As a result, current PD management remains fundamentally symptomatic, with emphasis on optimizing long-term function, minimizing treatment-related harm, and preserving participation rather than pursuing unproven neuroprotective strategies [62]. This pragmatic orientation reflects the present evidentiary landscape rather than a lack of biological ambition.

### 7.3. Multidisciplinary Care and Neurorehabilitation as Disease-Stabilizing Strategies

Multidisciplinary care refers to coordinated management delivered by multiple health professionals addressing complementary aspects of disease. In PD, this model is not ancillary but central to effective treatment, given the disorder’s multisystem involvement and evolving symptom profile.

Neurorehabilitation targets domains that pharmacological therapy cannot adequately modify. High-quality evidence supports physiotherapy for gait, balance, and fall prevention; occupational therapy for activities of daily living and environmental adaptation; speech and language therapy for hypophonia and dysphagia; and cognitive and psychological interventions for executive dysfunction and mood disorders [65,66]. These interventions yield reproducible functional benefits, often independent of changes in motor severity scales.

Escalation of pharmacological therapy in isolation frequently produces diminishing returns. Effective treatment, therefore, requires continuous integration of medication management with rehabilitation, psychosocial support, and caregiver engagement, a principle increasingly emphasized in comprehensive PD care models [62].

### 7.4. Pharmacological Treatment of Motor Symptoms: Beyond Levodopa Responsiveness

#### 7.4.1. Levodopa as the Therapeutic Foundation

Levodopa remains the most effective treatment for motor symptoms in PD and continues to serve as the pharmacological foundation across disease stages. A sustained response supports diagnostic accuracy, whereas poor responsiveness raises concern for alternative parkinsonian syndromes [67].

With disease progression, the brain’s capacity to buffer fluctuations in dopamine levels diminishes, leading to motor fluctuations characterized by alternating ON and OFF states. These fluctuations reflect both disease-related loss of presynaptic buffering capacity and the pharmacokinetic limitations of intermittent oral dosing [62].

#### 7.4.2. Adjunctive Therapies and the Limits of OFF-Time Reduction

Adjunctive pharmacological therapies aim to reduce OFF time by prolonging levodopa availability or stimulating dopaminergic pathways through complementary mechanisms. As summarized in Table 2, high-certainty evidence supports extended-release levodopa formulations, dopamine agonists (notably pramipexole), opicapone, rotigotine, and safinamide in reducing daily OFF time by approximately one hour or more [68,69,70].

Across trials, however, improvements in functional capacity and quality of life are modest, variable, or absent, despite statistically significant motor benefits. Dopamine agonists demonstrate the most consistent functional effects but are constrained by neuropsychiatric and behavioural adverse events, including impulse-control disorders and somnolence [71]. Moderate-certainty evidence supports monoamine oxidase–B inhibitors, catechol-O-methyltransferase inhibitors, zonisamide, and non-dopaminergic agents such as istradefylline, which provide incremental symptomatic benefit but rarely alter disability trajectories [62].

These findings reinforce a central principle: reduction of OFF time is necessary but insufficient as a surrogate for meaningful long-term outcomes. Adjunctive therapies should therefore be viewed as tools for symptom optimization rather than definitive solutions for advanced disease.

#### 7.4.3. Continuous Dopaminergic Delivery: Functional Relevance over Motor Metrics

Continuous dopaminergic delivery aims to minimize plasma dopamine fluctuations by providing stable stimulation over extended periods [62]. This strategy directly addresses the limitations of intermittent oral therapy.

As detailed in Table 1, levodopa–carbidopa intestinal gel, continuous subcutaneous apomorphine infusion, and subcutaneous foslevodopa–foscarbidopa achieve larger and more consistent reductions in OFF time than oral adjunctive therapies. Notably, levodopa–carbidopa intestinal gel has demonstrated clinically meaningful improvements in functional outcomes and quality of life, extending beyond reductions in motor scores [72].

Beyond motor smoothing, continuous delivery may reduce fluctuation-related anxiety and facilitate engagement in rehabilitation and daily activities. Nevertheless, invasiveness, device-related complications, and substantial infrastructure requirements limit scalability and equitable access, particularly outside specialized centres [62].

#### 7.4.4. Non-Motor Symptoms: Central Determinants of Patient Experience

Non-motor symptoms—including cognitive impairment, mood and anxiety disorders, psychosis, autonomic dysfunction, sleep disturbances, fatigue, and pain—often evolve independently of motor impairment and represent the primary determinants of quality of life in PD [7,50].

Pharmacological treatment remains symptom-specific and largely palliative. Acetylcholinesterase inhibitors provide modest benefit for cognitive impairment; antidepressants for mood disorders; pimavanserin or selected atypical antipsychotics for psychosis; and targeted therapies for autonomic and sleep-related symptoms [62]. Pain remains under-recognized and undertreated, with limited high-quality evidence guiding management beyond dopaminergic optimization and multidisciplinary approaches.

Failure to systematically address non-motor symptoms is a major contributor to perceived treatment failure, even among patients with apparently adequate motor control.

#### 7.4.5. Surgical and Device-Aided Therapies: High Efficacy, High Selectivity

Deep-brain stimulation (DBS) represents the most established surgical therapy for PD. As summarized in Table 3, high-certainty evidence supports globus pallidus internus stimulation in patients with levodopa-responsive motor symptoms complicated by disabling fluctuations or dyskinesias. DBS produces sustained improvements in motor function, functional capacity, and quality of life but does not alter disease progression and has a limited impact on non-motor symptoms [73,74].

Unilateral pallidotomy retains limited relevance where DBS is unavailable, while other ablative or experimental approaches—including neurotrophic factor delivery—remain investigational due to inconsistent efficacy.

#### 7.4.6. Access, Equity, and the Gap Between Evidence and Practice

A defining challenge in PD treatment is the persistent disconnect between therapeutic efficacy and real-world availability. As highlighted in Table 2 and Table 3, many of the most effective pharmacological and device-aided therapies remain inaccessible in low- and middle-income settings due to regulatory, economic, and infrastructural barriers [62].

Effective treatment of PD must therefore be conceptualized as a systems-level challenge, requiring the integration of evidence-based interventions, neurorehabilitation capacity, specialist expertise, and health-system infrastructure to translate scientific advances into durable, population-level benefits [1,62].

## 8. Discussion

The future of PD research and care depends on strategies that move beyond exclusive reliance on late-stage symptomatic management toward prevention, earlier biological definition, and more rationally targeted intervention. At present, however, the gap between expanding biological insight and tangible clinical transformation remains substantial. A critical long-term objective is to prevent PD through coordinated efforts that address upstream determinants of risk, including environmental toxicant exposure, lifestyle factors, and social inequities. Reducing exposure to pesticides and industrial solvents, alongside promotion of physical activity and cardiovascular health, represents a plausible population-level approach to lowering disease incidence, even in the absence of individual-level biological stratification. Nevertheless, implementation remains uneven across regions and socioeconomic strata [9].

Expanding genetic research to include historically underrepresented populations constitutes both a scientific necessity and an ethical imperative. Current genetic knowledge is disproportionately derived from individuals of European ancestry, limiting generalisability and constraining the equitable application of precision medicine. Recent identification of population-specific risk variants underscores that genetic architecture is neither uniform nor globally transferable [40]. Without deliberate diversification of genetic and biomarker cohorts, advances in stratified therapies risk reinforcing existing disparities rather than alleviating them.

Technological innovation is poised to reshape PD care but introduces new layers of complexity. Artificial intelligence–driven analytics, digital biomarkers, and wearable technologies offer opportunities for earlier detection, longitudinal monitoring, and individualized treatment adjustment [75]. However, their clinical utility is constrained by variable data quality, lack of validated functional endpoints, regulatory uncertainty, and unequal access to digital infrastructure. Telemedicine and remote monitoring may mitigate geographic barriers, yet their benefits are unevenly distributed, particularly in low- and middle-income settings where advanced diagnostics, infusion therapies, and device-aided treatments remain largely inaccessible [1,62].

The development of reliable biological biomarkers remains a central, unmet need. α-Synuclein seed amplification assays demonstrate high sensitivity and specificity for synucleinopathies and offer unprecedented potential to identify PD at prodromal or preclinical stages [25,27]. Nevertheless, biomarker positivity currently defines biological presence rather than clinical destiny. Major limitations persist, including incomplete longitudinal validation, uncertain prognostic value, and a lack of consensus on how biomarker status should inform individual clinical decisions. Critically, well-validated intermediate endpoints linking biomarker modulation to durable functional outcomes are lacking, a gap that continues to undermine efforts toward disease-modifying therapy. The repeated failure of disease-modifying trials likely reflects late intervention, reliance on clinically defined populations, and insufficient biological stratification [9].

From a therapeutic perspective, this review highlights the persistent challenge of translating efficacious interventions into scalable, durable solutions. Continuous dopaminergic delivery systems and deep brain stimulation are supported by robust evidence of motor and functional benefits in selected patients, yet their real-world impact is constrained by cost, infrastructure requirements, and limited availability outside specialized centres [1,62]. Moreover, many clinical trials exclude older individuals, patients with cognitive impairment, and those from resource-limited settings, thereby limiting external validity and real-world relevance.

Taken together, these observations argue for a recalibration of priorities in PD research and care. Progress will depend less on incremental symptomatic optimization and more on integrating biological definition with functional outcomes, multidisciplinary care models, and implementation-aware trial design. Equally important is the recognition that prevention, environmental regulation, and health-system capacity represent core components of precision medicine at the population level, rather than competing or secondary strategies.

## 9. Conclusions

PD is a complex and heterogeneous neurodegenerative disorder characterized by progressive motor and non-motor manifestations that impose an escalating burden on individuals, healthcare systems, and societies worldwide. Although substantial advances have been made in elucidating genetic susceptibility, environmental contributors, and disease biology, current treatments remain predominantly symptomatic and do not alter the underlying neurodegenerative process.

Recent progress in biomarker discovery, genetic stratification, and advanced therapeutic technologies provides a credible pathway toward earlier biological definition and more rational clinical trials. However, precision therapy in PD remains aspirational rather than realized, constrained by late intervention, limited predictive power of current biomarkers, incomplete linkage between biological change and functional benefit, and profound inequities in access to care [1,62].

The next phase of PD management must therefore prioritize prevention, probabilistic biological classification, and equitable implementation. Achieving this will require coordinated global investment, inclusive research practices, and health-system innovation to ensure that advances in science translate into meaningful benefits across diverse populations. Only by aligning biological discovery with functional relevance and real-world accessibility can the field transition from managing PD to genuinely altering its trajectory.

## Figures and Tables

**Table 1:** Non-motor symptoms of Parkinson’s disease.

Domain	Symptom	Typical Timing *	Principal Pathophysiological Associations	Clinical Relevance
Autonomic	Constipation	Prodromal	Enteric α-synuclein pathology; vagal dysfunction	Common early feature; nonspecific in isolation
	Orthostatic hypotension	Early–Late	Sympathetic denervation; baroreflex failure	Falls, syncope, and increased mortality
	Urinary dysfunction	Early–Late	Pontine–autonomic network involvement	Sleep disruption; reduced quality of life
	Sexual dysfunction	Early	Autonomic and hypothalamic involvement	Psychosocial impact
	Sialorrhea	Early	Impaired oropharyngeal motor control	Aspiration risk
	Dysphagia	Early–Late	Brainstem motor nucleus involvement	Pneumonia; mortality
	Thermoregulatory dysfunction	Early–Late	Hypothalamic and autonomic dysfunction	Heat intolerance
Sensory	Hyposmia	Prodromal	Olfactory bulb and limbic Lewy pathology	Strong risk marker when combined with other features
	Visual disturbances	Early	Retinal dopaminergic loss; cortical dysfunction	Falls; hallucinations
	Pain	Prodromal–Late	Central and peripheral nociceptive dysfunction	Major determinant of disability
Sleep-related	REM sleep behaviour disorder	Prodromal	Brainstem REM atonia network failure	High predictive value for synucleinopathy
	Insomnia/sleep fragmentation	Early–Late	Brainstem and circadian network disruption	Cognitive and mood consequences
	Excessive daytime sleepiness	Early–Late	Hypothalamic dysfunction; medication effects	Accident risk
Neuropsychiatric	Depression	Prodromal–Late	Limbic and monoaminergic network dysfunction	Reduced quality of life
	Anxiety	Prodromal–Late	Amygdala–salience network dysfunction	Exacerbates motor fluctuations
	Apathy	Early–Late	Mesocorticolimbic circuit dysfunction	Predictor of cognitive decline
	Visual hallucinations	Early–Late	Cholinergic and visual network dysfunction	Dementia risk
Cognitive	Executive dysfunction	Prodromal	Frontostriatal network dysfunction	Early cognitive impairment
	Parkinson’s disease dementia	Late	Cortical α-synuclein ± mixed pathology	Major determinant of mortality

* Typical timing reflects longitudinal and clinicopathological observations; symptoms may occur outside these windows.

**Table 2:** Pharmacological treatments for motor fluctuations in Parkinson’s disease.

Treatment	Evidence Certainty *	Mean OFF-Time Reduction	Effect on Motor Severity	Functional Outcome (UPDRS-II/ADL)	Quality of Life	Key Limitations	Availability in LMICs ^†^
Levodopa–carbidopa ER (IPX066)	High	~1.2 h/day	Moderate improvement	Small (below MCID)	No meaningful change	Dyskinesia risk; cost	Rare
Opicapone	High	0.7–2.0 h/day	Improves ON time	Minimal	No consistent benefit	Diarrhea; cost	Limited
Pramipexole (IR/ER)	High	≥1 h/day	Moderate	Clinically relevant	Variable	Impulse-control disorders; somnolence	Wide
Rotigotine (patch)	High	≥1 h/day	Moderate	Variable	Inconsistent	Skin reactions; hallucinations	Limited
Safinamide	High	~1 h/day	Mild–moderate	Inconsistently assessed	Inconsistent	Limited non-motor benefit	Limited
Rasagiline	Moderate	0.5–0.9 h/day	Mild	Small (below MCID)	Small	Symptomatic only	Wide
Ropinirole (IR)	Moderate	~1 h/day	Moderate	Modest	Inconsistent	Behavioral adverse effects	Wide
Zonisamide	Moderate	0.7–1.4 h/day	Mild	Modest	Not assessed	Regional approval	Regional
Entacapone	Moderate	0.6–1 h/day	Mild	Minimal	Insufficient evidence	Frequent dosing	Wide
Istradefylline	Moderate	0.7–1 h/day	Mild	Limited	Not assessed	Cost: modest effect	Limited
Amantadine ER	Moderate	Modest	Dyskinesia reduction	Not assessed	Not assessed	Cognitive adverse effects	Rare
LCIG	Moderate	~1.9 h/day	Sustained	Clinically meaningful	Clinically meaningful	Invasive; device complications	Not available
Foslevodopa–foscarbidopa (SC)	Moderate	~1.8 h/day	Sustained	Not assessed	Not assessed	Emerging therapy	Not available
Apomorphine infusion	Moderate	~1.9 h/day	Sustained	Modest	No sustained benefit	Nausea; skin nodules	Select centres
Levodopa–carbidopa CR	Low	Inconsistent	Minimal	No improvement	No benefit	Unreliable kinetics	Wide
Selegiline	Low	Inconsistent	Minimal	No sustained benefit	No benefit	Limited efficacy	Wide
Nicotine (patch)	Very low	Uncertain	Uncertain	Not assessed	Not assessed	Not therapeutic	Not used
Terguride	Very low	None	None	None	Not assessed	Ineffective	Not available
Perampanel	Very low	None	None	None	Not assessed	Ineffective	Not available

* Evidence certainty assessed using GRADE methodology. ^†^ Availability reflects inclusion in national formularies and market access across Latin American countries.

**Table 3:** Surgical and device-aided interventions for motor fluctuations.

Intervention	Evidence Certainty *	Motor Benefit	Functional Outcome	Quality of Life	Key Safety Considerations	Availability in LMICs
GPi Deep Brain Stimulation	High	Large, sustained	Clinically meaningful	Clinically meaningful	Favorable cognitive profile	Limited
Unilateral pallidotomy	Moderate	Sustained (unilateral)	Modest	Modest	Irreversible; lesion effects	Select
Subthalamotomy	Low	Modest	Not assessed	Not assessed	Hemiballismus risk	Rare
Zona incerta DBS	Very low	Inconsistent	No clear benefit	No clear benefit	High bias; adverse events	Not available
Intraputaminal GDNF	Very low	None	None	None	Safe but ineffective	Experimental

* Based on randomized trials and high-quality observational data.

## Data Availability

No new data were created or analyzed in this study. Data sharing does not apply to this article.

## Abbreviations

The following abbreviations are used in this manuscript:

ADL	Activities of daily living
AI	Artificial intelligence
CSF	Cerebrospinal fluid
DBS	Deep brain stimulation
DJ1	Parkinson’s disease protein 7
DLB	Dementia with Lewy bodies
ER	Extended release
GBA1	Glucocerebrosidase 1
GDNF	Glial cell line–derived neurotrophic factor
IR	Immediate release
LCIG	Levodopa–carbidopa intestinal gel
LMICs	Low- and middle-income countries
LRRK2	Leucine-rich repeat kinase 2
MCI	Mild cognitive impairment
MCID	Minimal clinically important difference
MDS	Movement Disorder Society
MRI	Magnetic resonance imaging
OFF time	Periods of reduced motor function
ON time	Periods of optimal motor function
PD	Parkinson’s disease
PET	Positron emission tomography
PINK1	PTEN-induced kinase 1
PRKN	Parkin
RBD	Rapid eye movement sleep behaviour disorder
REM	Rapid eye movement
SNCA	α-synuclein gene
SPECT	Single-photon emission computed tomography
UPDRS	Unified Parkinson’s Disease Rating Scale
